# Association between Clinical Frailty Scale and mortality 24 months after hospitalisation in adult patients with COVID-19

**DOI:** 10.1016/j.heliyon.2024.e40456

**Published:** 2024-11-15

**Authors:** Julia Minnema, Melvin Lafeber, Roos S.G. Sablerolles, Janneke A.L. van Kempen, Lisanne Tap, Harmke A. Polinder-Bos, Bob P.A. van de Loo, Hugo van der Kuy, Miriam C. Faes, on behalf of the COMET-, and COOP-research group, Jacomien Aleman, Jos Tournoy, Lorenz Van der Linden, Marco Gambera, Isabella Martignoni, Ronald Van Etten, Hein van Onzenoort, Mariette Kappers, Peter van Wijngaarden, Jose Verstijnen, Vera Theeuwes, Marleen Kemper, Elise Slob, Ferdi Sombogaard, Heshu Abdullah-Koolmees, Roland van den Berg, Hugo de Wit, Betul Dilek, Freija Hogenhuis, Vahid Buyukayten, Britt te Brake, Margriet Nieuwenhuijzen, Maria Scheeren, Madelief de Wit, Arjan Bulsink, Ingrid van Haelst, Peter ter Horst, Rosalie Moorlag, Anja Vos, Annemiek Otten-Helmers, Erik van Kan, Marije Voskamp, Marieke Ebbens, Marieke Ezinga, Cees van Nieuwkoop, Loes Visser, Caroline Ghazarian, Doranne Hilarius, Gonneke Hermanides, Carlinda Bresser, Judith Derijks-Engwegen, Ebbie Boemaars, Zahira Getrouw, Barbara Maat, Peter Wierenga, Tessa Bosch, Lisanne Krens, Kajie Liang, Langeza Saleh, Milou van Heuckelum, Linda Hendriksen, Paul van der Linden, Kaylen Guda, Kristel Crommentuijn, Ilse Cornelissen-Wesseling, Jeroen Diepstraten, Jacobien Ellerbroek, Saskia Coenradie, Debbie Deben, Kim Hurkens, Dennis Wong, Marion Vromen, Marjolein de Bock, Suzan Savelkoul, Saskia Wolters, Louise Andrews, Eefje Jong, Rosanne Kranenburg, Joana Soares, Fatima Falcao, Mariana Solano, Erica Viegas, Margarida Falcao, Helena Farinha, Dina Mendes, Joao Rijo, Marta Miarons, Maria Queralt Gorgas, Cristina García Yubero, Laura Portillo Horcajada, Kim Keijzers, Silke Lim, Linden Ashfield, Helen Bell, Naomi Fitzhugh, Glenda Fleming, Nicola Goodfellow, Joanne Hanley, Michael Scott, Simon P. Mooijaart, Jacobijn Gussekloo, Petra Elders, Geeske Peeters

**Affiliations:** a Department of Internal Medicine, Erasmus MC, University Medical Centre Rotterdam, the Netherlands; b Department of Hospital Pharmacy, Erasmus MC, University Medical Centre Rotterdam, the Netherlands; c Department of Geriatrics, Amphia Hospital, Breda, the Netherlands; d Digitalis Rx BV, Amsterdam, the Netherlands

**Keywords:** Frailty, COVID-19, Long-term, Survival

## Abstract

**Background:**

The clinical frailty scale (CFS) was used as a triage tool for medical decision making during the COVID-19 pandemic. The CFS has been posed as a suitable risk marker for in-hospital mortality in COVID-19 patients. We evaluated whether the CFS is associated with mortality 24 months after hospitalisation for COVID-19.

**Methods:**

The COvid MEdicaTion (COMET) study is an international, multicentre, observational cohort study, including adult patients hospitalised for COVID-19 between March 2020–July 2020. Patients’ characteristics, prescribed medication, clinical characteristics, and CFS were collected at admission, survival data were collected 24 months after hospitalisation. Multivariable cox proportional hazard models stratified by age (<65 and ≥65 years), and adjusted for covariates (age, sex, number of drugs, and types of drug class as a proxy for comorbidities) were used to study the association between the CFS and 24-month mortality after hospitalisation.

**Results:**

In this study, 1238 fit (CFS 1–3), 478 mildly frail (CFS 4–5), and 235 frail (CFS 6–9) patients were included for baseline analysis (median age 68 years (IQR 58–78); 58.5 % male). Frailty was associated with an increased risk of 24-month mortality after hospitalisation in older patients (HR 1.91, 95 % CI [1.17–3.12]), in younger adults a trend was seen (HR 3.13, 95 % CI [0.86–11.36]).

**Conclusion:**

The results suggest that the CFS is an indicator for mortality 24 months after hospitalisation in COVID-19 patients.

## 1 Introduction

The large number of critically ill COVID-19 patients hospitalised during the first wave of the COVID-19 pandemic overwhelmed many healthcare systems. The scarcity of resources, especially intensive care unit (ICU) capacity, necessitated the development and implementation of triage guidelines. The use of reliable clinical parameters is essential to perform triage adequately [1,2]. In this context, the interest and need to define the degree of frailty has grown rapidly. Frailty is a condition characterised by a decline in multiple physiological systems and increased vulnerability to stressors, and is related to adverse health outcomes, such as functional decline, loss of independence and mortality [[3], [4], [5]].

Before the COVID-19 pandemic, triage decisions for critical care were often made without considering frailty. During the pandemic, explicit consideration of frailty has become more common through the use of the Clinical Frailty Scale (CFS). This is a clinical judgement-based tool designed to summarise the level of fitness of older patients two weeks before hospital admission [3,6,7]. The CFS is based on the principle of accumulation of age-associated health deficits, and is validated in older adults [3]. However, in younger patients, disabilities usually reflect a single deficit, and it is unknown whether the CFS can detect this specific type of frailty [7]. A few studies before the pandemic have extended the use of the CFS in patients younger than 65 years old [[8], [9], [10]]. For instance, De Geer et al. showed that the CFS was associated with 180-day mortality in ICU patients aged 50–64 years old [10]. Moreover, the study of Darvall et al. showed that only CFS 7–8 were associated with mortality in non-COVID-19 pneumonia in patients aged 16 years and older [9].

Recently, the CFS has been proposed as an indicator for in-hospital mortality in COVID-19 patients [8]. Thus far, little is known about the association between frailty and long-term survival after hospitalisation for COVID-19. Multiple studies suggest a relationship between the CFS and mortality 6 months and 1 year after hospitalisation, but longer follow-up durations remain to be evaluated [11,12]. Knowledge about the long-term impact of COVID-19 on mortality can aid clinicians in medical decision making regarding the allocation of resources, especially in times of scarcity. Specifically older adults often face such difficult decisions due to their higher risk on severe disease and short-term mortality. Hence, this study aims to investigate the association between the CFS and 24-months mortality after hospitalisation in adult COVID-19 patients younger than 65 years and 65 years and older.

## 2 Methods

### 2.1 Study design and participants

The COvid MEdicaTion (COMET) study is an international, multicentre observational cohort study. Baseline data has been collected in 63 hospitals in 11 European countries. The inclusion criteria comprised patients aged 18 years and older who were hospitalised due to SARS-CoV-2 infection. The patients were included during the first wave of the COVID-19 pandemic (March–July 2020). The rationale and design of the COMET study have previously been described in detail [13]. In early 2022, the participating centres were asked to collect data for this COMET follow-up study. Patients who deceased during hospitalisation, did not have an available baseline CFS score or did not participate in the follow-up study were excluded. Patients who survived hospital admission with an available CFS and follow-up data were eligible for analyses. In total, 30 hospitals from 6 European countries (Belgium, Italy, the Netherlands, Portugal, Spain, Switzerland) participated. The members of the COMET-, and COVID-19 Outcomes in Older People (COOP) research groups are listed in Supplemental Tables 1 and 2 This study is not subject to the Research Involving Human Subjects Act (WMO), due to the descriptive nature of the study. The medical ethics committees of all hospitals waived the necessity for formal approval of the study. All data were treated according to the European privacy regulations and the study was done in accordance with the declaration of Helsinki.

### 2.2 Data collection

The frailty status of the participants was assessed with the CFS. Participants were categorised in three groups: fit (CFS 1–3), mildly frail (CFS 4–5) or frail (CFS 6–9). Participants had their CFS recorded prospectively on the first day of admission or retrospectively by trained researchers based on available data in medical records. Retrospective assessment of CFS has been clinically validated in multiple studies [14,15]. In addition, the CFS was found to be a reliable measure with a high inter-rater reliability [16]. The survival data for this follow-up study were retrospectively collected from the electronic medical records between May 2022–January 2023, approximately 24 months after the patients were discharged from the hospital [13]. The follow-up period is defined as 24 months after hospital discharge. We collected the date of hospital admission and discharge, ICU admission (if applicable: date of ICU admission and discharge, and the use of ventilator assisted breathing), survival status 24 months after hospital discharge (if applicable: date of death), and destination of discharge. In order to ensure a homogeneous definition of ICU beds, the principal investigators were approached by e-mail to clarify whether their hospital had intermediate care beds and if they were designated as an ICU bed. In this study, an intermediate care bed was not considered an ICU bed.

Data were collected in an online database (Clinical Rules reporter, version 1.6.3; Digitalis Rx, Amsterdam, the Netherlands). Patients will be entered into the database with a code. No data that can identify a patient will be processed on this database to protect and respect the privacy of the patients. The participant's study number of the original study was used [13]. The encoding file was only available to the local investigator.

### 2.3 Outcomes

The primary outcome for current analyses was mortality within 24 months after hospital discharge for COVID-19, excluding in-hospital mortality.

### 2.4 Statistical analysis

Descriptive analyses were performed for the characteristics of patients after hospital discharge (excluding in-hospital mortality), stratified by CFS category and age (<65 years vs ≥65 years). The three categories of CFS were defined as fit (CFS 1–3), mildly frail (CFS 4–5) and frail (CFS 6–9) (14). Fit patients were used as the reference category. Differences in baseline characteristics between the three CFS categories for the two age groups separately were analysed by using a one-way ANOVA for continuous variables and extended Chi-square test for categorical variables.

The primary outcome was evaluated using Kaplan-Meier curves. Additionally, Cox Proportional Hazards Models were derived, which were stratified by age (<65 years vs ≥65 years) and adjusted for covariates at hospital admission. Four different models were built.

• Model 1: A crude, unadjusted estimate
• Model 2: Model 1 and an additional adjustment for age and sex
• Model 3: Model 2 and additional adjustment for the number of drugs used by the patient
• Model 4: Model 3 and further adjustment for concomitant drugs

To determine if the association between CFS and 24-month mortality was different for patients <65 years and patients ≥65 years of age, we used a multiplicative interaction term to provide statistical evidence whether effect sizes between the age groups were different or not. In addition, to determine if the association between CFS and 24-month mortality was different for men and women, we used a multiplicative interaction term to provide statistical evidence whether effect sizes differed between men and women. Additionally, a Kaplan-Meier curve and similar Cox Proportional Hazard Models for 24-months mortality after hospital admission, but including in-hospital mortality, were built. Estimates were presented as hazard ratios with 95 % confidence intervals (CI).

Three sensitivity analyses were performed. The first sensitivity analysis compared the sample within the follow-up study with and without a CFS. Because the CFS was not scored for all enrolled patients in the follow-up study, characteristics of the subgroup of patients with a CFS were compared with the subgroup of patients with a missing CFS. Statistically significant differences in characteristics were examined using the Mann-Whitney *U* test for continuous variables and the Chi-square test for categorical variables. The second sensitivity analysis compared characteristics of the study sample in the Netherlands with other participating European countries, since the number of included patients in the Netherlands was significantly higher. Statistically significant differences in characteristics were examined using the Mann-Whitney *U* test for continuous variables and X^2^ test for categorical variables. The third sensitivity analysis examined the association between the CFS and 24-months mortality after hospitalisation (excluding in-hospital mortality) using a multivariable logistic regression analysis resulting in an odds ratio, since this analysis is less sensitive compared to a cox regression analysis. The same four models were built as the primary regression analysis.

The analyses were performed using SPSS version 28.0.1.0.

## 3 Results

Baseline and follow-up data were collected for 2879 patients between May 2022 and January 2023. Patients who died in hospital (n = 421) or had a missing CFS (n = 507) were not included in the main analysis. The remaining patients (n = 1951) were included in the baseline and outcome analyses (Belgium n = 73, Switzerland n = 5, Italy n = 40, the Netherlands n = 1619, Portugal n = 118, and Spain n = 96).

Baseline characteristics for the included patients were compared and are presented in Table 1 (p-values between groups are listed in Supplemental Table 3). Among the patients ≥65 years, frail patients were older (median 84 [IQR 75–90] vs. median 74 [69–79]) and less often admitted to the ICU (13.1 % vs. 25.6 %) compared to fit patients. Furthermore, frail patients were less often discharged back to home compared to fit patients (42.1 % vs. 73.2 %). Among patients <65 years, frail patients were prescribed more drugs (median 4 [[2], [3], [4], [5], [6], [7], [8], [9]] vs. 1 [[1], [2], [3], [4]]) compared to fit patients.

**Table 1:** Patient characteristics after hospital discharge (excluding in-hospital mortality) stratified by CFS category and age (n = 1951).

	Age <65 years n = 843 (43.2 %)			Age ≥65 years n = 1108 (56.8 %)		
	CFS 1–3 n = 676 (80.2 %)	CFS 4–5 n = 112 (13.3 %)	CFS 6–9 n = 55 (6.5 %)	CFS 1–3 n = 562 (50.7 %)	CFS 4–5 n = 366 (33 %)	CFS 6–9 n = 180 (16.2 %)
**Patient characteristics**						
Age (years)	56 [47–61]	60 [55–63]	57 [52–62]	74 [69–79]	79 [74–84]	84 [75–90]
Men	401 (59.3)	67 (59.8)	32 (58.2)	346 (61.6)	204 (55.7)	91 (50.6)
**Use of medication pre-admission**						
Blood pressure-lowering drugs	150 (22.2)	46 (41.1)	21 (38.2)	318 (56.6)	248 (67.8)	128 (71.1)
Antiplatelet drugs	38 (5.6)	17 (15.2)	9 (16.4)	119 (21.1)	127 (34.7)	56 (31.1)
Oral anticoagulant drugs	13 (1.9)	3 (2.7)	4 (7.3)	79 (14.1)	64 (17.5)	49 (27.2)
Glucose-lowering drugs	66 (9.8)	25 (22.3)	8 (14.5)	105 (18.7)	97 (26.5)	48 (26.7)
Antipsychotic drugs and cholinesterase inhibitors	9 (1.3)	4 (3.6)	2 (3.6)	13 (2.3)	13 (3.6)	23 (12.8)
Number of prescribed drugs	1 [1–4]	5 [1–8]	4 [2–9]	4 [2–7]	7 [4–10]	8 [5–11]
**In-hospital outcomes**						
Length of stay hospital (days)	5 [3–12]	5 [3–8]	8 [4–21]	7 [4–15]	6 [3–10]	8 [4–15]
Intensive care admission	159 (25.0)	22 (19.6)	23 (41.8)	142 (25.6)	51 (14.0)	23 (13.1)
Length of stay ICU (days)	9 [3–18]	6 [3–14]	9 [6–18]	11 [2–22]	4 [1–12]	9 [3–13]
Ventilator assisted breathing	124 (78.0)	18 (81.8)	19 (82.6)	111 (78.2)	36 (70.6)	19 (82.6)
**Destination at discharge**						
Home	521 (89.8)	83 (90.2)	37 (71.2)	360 (73.2)	187 (62.5)	69 (42.1)
Nursing home	3 (0.5)	0 (0)	2 (3.8)	14 (2.8)	17 (5.7)	33 (20.1)
(Geriatric) rehabilitation	56 (9.7)	9 (9.8)	13 (25.0)	118 (24.0)	95 (31.8)	62 (37.8)
**Long-term outcomes**						
24-months mortality	10 (1.5)	10 (8.9)	4 (7.3)	46 (8.2)	59 (16.1)	49 (27.2)

P-values: provided in Supplemental Table 3.

Data are presented as median [IQR] for skewed variables, and N (percentages) for categorical variables.

**Fig. 1:**
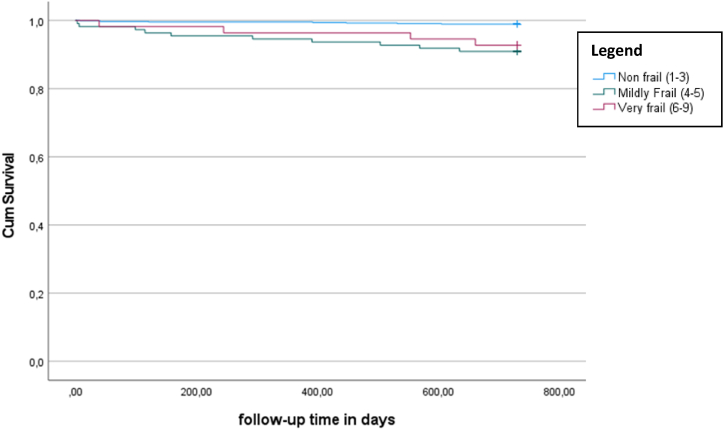
24-months mortality after hospital discharge for patients <65 years of age (excluding in-hospital mortality) (n = 800).

**Fig. 2:**
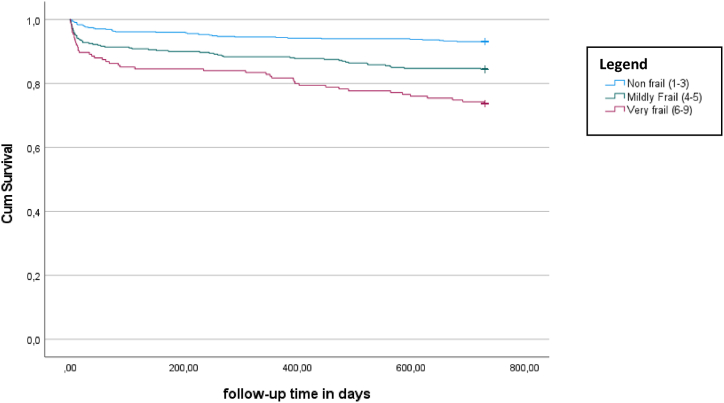
24-months mortality after hospital discharge for patients ≥65 years of age (excluding in-hospital mortality) (n = 1085).

The Kaplan-Meier curves for survival 24-months after hospital discharge for patients <65 years of age and patients ≥65 years are shown in Fig. 1, Fig. 2, respectively. The average survival time after hospital discharge for patients <65 years was for fit patients 725.3 days, for mildly frail patients 688.8 days, and for frail patients 704.2 days, with an overall-survival time of 718.9 days. The difference in survival distributions for the three CFS groups was statistically significant (ꭓ^2^ = 29.4, p < 0.01). The average survival time after hospital discharge for patients ≥65 years was for fit patients was 692.5 days, for mildly frail patients 643.8 days, and for frail patients ≥65 years 594 days, with an overall-survival time of 660.4 days. The difference in survival distributions for the three CFS groups was statistically significant (ꭓ^2^ = 49.2, p < 0.01).

The association between CFS and 24-months mortality post-discharge is presented in Table 2. After adjustment for covariates (model IV), older frail patients had an increased risk of mortality 24-months after hospital discharge compared to older fit patients (HR 1.91 [95 % CI 1.17–3.12]), whereas for mildly frail patients not statistically significant result was observed (HR 1.48 [95 % CI 0.96–2.27]). Contrary, in the sample of younger patients, frail patients did not have an significantly increased risk of mortality 24-months after hospital discharge compared to fit patients (HR 3.13 [95 % CI 0.86–11.36]), whereas mildly frail patients have an increased risk of mortality 24-months after hospital discharge compared to fit patients (HR 4.43 [95 % CI 1.60–12.25]). The multiplicative interaction between CFS and age was not statistically significant (p = 0.348), which showed that the association between CFS and 24-months mortality was not significantly different between age groups. In addition, the multiplicative interaction between CFS and sex was not statistically significant in patients <65 years of age (p = 0.644) and in patients ≥65 years of age (p = 0.354), which shows that the association between CFS and 24-months mortality was not significantly different between men and women. The association between CFS and 24-months mortality post-discharge is also presented in Supplemental Table 4, which included the values for the confounders included in model 4.

**Table 2:** Cox proportional hazard analysis for CFS and 24-months mortality post hospital discharge (n = 1885).

	CFS 1–3	CFS 4–5	CFS 6–9
Age <65 years (n = 800, number of events = 21)			
Model 1	Ref.	8.61 (3.28–22.63)	6.75 (1.98–23.07)
Model 2	Ref.	6.51 (2.44–17.37)	5.91 (1.72–20.33)
Model 3	Ref.	3.89 (1.35–11.23)	3.72 (1.05–13.23)
Model 4	Ref.	4.43 (1.60–12.25)	3.13 (0.86–11.36)

Age ≥65 years (n = 1085, number of events = 140)			
Model 1	Ref.	2.38 (1.57–3.59)	4.23 (2.75–6.50)
Model 2	Ref.	1.73 (1.13–2.65)	2.29 (1.42–3.70)
Model 3	Ref.	1.46 (0.95–2.24)	1.86 (1.15–3.02)
Model 4	Ref.	1.48 (0.96–2.27)	1.91 (1.17–3.12)

Estimates are hazard ratios (95 % CI). CFS=Clinical Frailty Scale. Model I = crude. Model II = adjusted for sex and age. Model III = model II plus additional adjustment for the number of drugs used. Model IV = model III plus additional adjustment for blood pressure-lowering drugs, antiplatelet drugs, oral anticoagulant drugs, glucose-lowering drugs, antipsychotic drugs, and cholinesterase inhibitors.

A sensitivity analysis was performed for the association between the CFS and 24-months mortality after hospital discharge using a logistic regression analysis and is presented in Supplemental Table 5. After adjustment for the predefined covariates (model IV), older frail patients had an increased risk for mortality 24 months after hospital discharge (OR 1.88 [95 % CI 1.13–3.15]), whereas for mildly frail this association was not found (OR 1.34 [95 % CI 0.86–2.08]). Contrary, in the sample of younger patients, mildly frail patients had an increased risk for mortality 24 months after hospital discharge (OR 3.65 [95 % CI 1.36–9.83]), whereas for younger frail patients a trend was seen (OR 2.71 [95 % CI 0.75–9.83]).

The patient characteristics for the total study sample (including in-hospital mortality) is presented in Supplemental Tables 6 and 7 The Kaplan-Meier curve for survival 24 months after hospital admission is shown in Supplemental Fig. 1. The average survival time after hospital admission for fit patients was 641.9 days, for mildly frail patients 496.1 days, and for frail patients 396.1 days. The difference in survival distributions for the three CFS groups was statistically significant (ꭓ^2^ = 282.0, p < 0.01). The association between CFS and 24-months mortality after hospital admission (including in-hospital mortality) is shown in Supplemental Table 8. After adjustment for covariates (model IV), older frail patients had an increased risk of mortality 24-months after hospital admission compared to older fit patients (HR 1.84 [95 % CI 1.44–2.34]). Similarly, older mildly frail patients had an increased risk of mortality 24-months after hospital admission compared to older fit patients (HR 1.41 [95 % CI 1.13–1.76]). Likewise, in the sample of younger patients, frail (HR 3.36 (95 % CI 1.54–7.33) and mildly frail (HR 3.23 95 % CI [1.65–6.33]) patients had an increased risk of mortality 24-months after hospital admission compared to older fit patients.

A sensitivity analysis was performed for patients of the COMET follow-up study in which the CFS was collected and where the CFS was missing and is shown is Supplemental Table 9. No statistically significant baseline differences were found between the CFS sample and non-CFS sample. However, the non-CFS sample showed a higher rate of in-hospital mortality (22.3 % vs. 17.7 %, p = 0.021), and a lower rate of 24-months mortality (68.9 % vs. 74.7 %, p = 0.008). Additionally, a sensitivity analysis was performed within the study sample of patients in the Netherlands and other countries as shown in Supplemental Table 10. Dutch patients were in general slightly older (mean 72 IQR [61–80] vs. 68 [54–79], p < 0.001) and were prescribed more drugs (mean 5 IQR [[2], [3], [4], [5], [6], [7], [8]] vs. 3 [[1], [2], [3], [4], [5], [6], [7]], p < 0.001). No differences between in-hospital mortality and 24-months mortality were found between the Netherlands and other countries.

## 4 Discussion

The aim of this study was to investigate the association between the CFS and 24-months mortality in patients <65 years and ≥65 years admitted to the hospital for COVID-19. The main findings of this study were twofold. Firstly, we found that frail older patients have a significantly higher risk of mortality 24 months after hospital discharge for COVID-19 compared to fit patients. For younger frail patients a trend was observed, although this was not statistically significant. The multiplicative interaction term showed no significant difference for the association between CFS and 24-month mortality between age groups and between men and women. Secondly, only a limited number of patients died after hospital discharge, whereas a significantly higher number died during hospital admission. In summary, our results suggest that the CFS is a suitable risk marker for short-term as well as long-term mortality in both younger and older patients admitted to the hospital for COVID-19.

The use of the CFS as a risk marker for short- and long-term mortality in older patients is in line with findings in literature. In the previous study of the COMET research group, the CFS was identified as a significant risk marker of hospital mortality in both younger (<65 years of age) and older (≥65 years of age) COVID-19 patients [8]. The current follow-up study extends this knowledge by indicating that the CFS is a risk marker of mortality 24-months post discharge in older patients. However, no significant association was found between the CFS and mortality 24-months post discharge in younger patients. In addition, in a large (n = 1344) Italian sample of COVID-19 patients with a mean age of 68 years old, a significant association (HR = 1.35, 95%CI 1.23–1.48) was found between frailty and in-hospital and medium-term mortality (median follow-up of 253 days) [17]. In a Brazilian study, which included severe COVID-19 patients admitted to the ICU, frailty was also found to be independently associated with long-term (6-months) mortality. However, we should be cautious when applying the CFS in younger people. Initially, the CFS was designed as a tool based on the accumulation of deficits, a natural occurrence among older people and not typical in younger people. In the study of de Geer and colleagues, the CFS was highly predictive for 180-day mortality after ICU admission for patients between 50 and 64 years [10]. This suggests that the CFS might also be useful in medical decision making in younger patients.

Our study has several limitations which need to be considered while interpreting the study results. Firstly, for some patients the CFS was collected retrospectively. However, research shows that retrospectively and prospectively collected CFS correspond strongly [15]. Furthermore, we did not collect a disease severity measure due to the pragmatic study design as described in the study protocol [13]. However, the CFS is a patient-specific clinical judgement tool with an explicit role in triage during the pandemic. Additionally, as some countries were represented by a few patients, the distribution and representation between northern and southern regions may not reflect reality. Due to this reason, it was not possible to build a multilevel model for the analysis of our data taking into account the treatment region. Adjustment for the regions could have made our model fit better compared to regression analysis due to the clustering nature of the data. However, for several countries we only had one centre and also limited patients from that centre. Moreover, the definition of an ICU bed is not generally defined in Europe, and can even differ within countries. However, we tried to overcome this limitation by not including intermediate care beds in our analyses.

Despite the previously mentioned limitations, this report was strengthened by the fact that patients from 30 centres and 6 European countries were included. Furthermore, we investigated the use of the CFS in patients younger than 65 years and we have assessed important parameters such as ICU admission and premorbid medication use.

In short, this study gives a clear view on the role of frailty during the COVID-19 pandemic and its association with long-term mortality. Nevertheless, certain factors remain unclear. More research is needed in additional waves of the pandemic to assess the association between the CFS and long-term mortality. However, knowledge on the use of CFS during the COVID-19 pandemic can help in multiple (pandemic) conditions. More research should investigate if the CFS predicts outcomes in conditions not limited in COVID-19 so that outcomes can effectively be altered by investigating targeted interventions. Also, important (clinical) parameters for older adults, such as quality of life and patient experiences, are not taken into account in this study. Additionally, more research is needed tailored towards the application of the CFS in younger patient groups.

To conclude, in the present study we observed an increased long-term mortality risk for older frail patients who have been hospitalised for COVID-19. These findings suggest that the CFS is a valuable tool which can be used during triage decisions for COVID-19 and to predict long-term outcomes. For future potential pandemics, it remains critical to assess each patient in a holistic way, not only taking into account age and (co-)morbidity, but also frailty status.

## Dissemination declaration

We have reported whether we plan to disseminate the results to study participants, patients organizations or stated that dissemination to these groups is not applicable.

## Data availability

The data underlying this article will be shared on reasonable request to the corresponding author.

## Ethics statement

This study is not subject to the Research Involving Human Subjects Act (WMO), due to the descriptive nature of the study. The institutional review committee of the main site, the Erasmus MC, approved the study (MEC-2020-0277), and each institutional review board of the participating hospitals approved the use of the data. Patients will be entered into the database with a code. No data that can identify a patient will be processed on this database to protect and respect the privacy of the patients.

## Declaration of competing interest

The authors declare the following financial interests/personal relationships which may be considered as potential competing interests:Hugo van der Kuy reports financial support was provided by 10.13039/501100001826Netherlands Organisation for Health Research and Development. Hugo van der Kuy reports financial support was provided by LOEY Foundation. If there are other authors, they declare that they have no known competing financial interests or personal relationships that could have appeared to influence the work reported in this paper.
