# Supplementary material for: Association between Clinical Frailty Scale and mortality 24 months after hospitalisation in adult patients with COVID-19
Source: Heliyon. 2024 Nov 15;10(23):e40456. doi: 10.1016/j.heliyon.2024.e40456 (PMC11648057; doi:10.1016/j.heliyon.2024.e40456)
Supplement: Multimedia component 1 [file mmc1.docx]

**SUPPLEMENTARY**

**Supplemental Table 1.** List of authors COMET research group

| **Aruba**  *Dr Horacio E Oduber hospitaal, Oranjestad:* Jacomien Aleman (PharmD) |
| --- |
| **Belgium**  *University Hospitals Leuven, Leuven:* Jos Tournoy (MD, PhD), Lorenz Van der Linden (PharmD) |
| **Italy**  *Pederzoli Hospital, Peschiera del Garda, VR:* Marco Gambera (PharmD), Isabella Martignoni (PharmD) |
| **The Netherlands**  *Amphia ziekenhuis, Breda:* Ronald Van Etten (MD), Hein van Onzenoort (PharmD), Mariette Kappers (MD), Peter van Wijngaarden (MD), Jose Verstijnen (BSc), Vera Theeuwes (MSc)  *Amsterdam UMC, AMC, Amsterdam:* Marleen Kemper (PharmD), Elise Slob (PharmD)  *Amsterdam UMC, VUmc, Amsterdam:* Ferdi Sombogaard (PharmD, PhD), Heshu Abdullah-Koolmees (PharmD), Roland van den Berg (PharmD)  *Canisius Wilhelmina Hospital, Nijmegen:* Hugo de Wit (PharmD)  *Erasmus Medisch Centrum, Rotterdam:* Betul Dilek (BSc), Freija Hogenhuis (PharmD), Vahid Buyukayten (BSc), Britt te Brake (BSc), Margriet Nieuwenhuijzen, Maria Scheeren, Madelief de Wit (BSc)  *Streekziekenhuis Koningin Beatrix Winterswijk:* Arjan Bulsink (PharmD)  *Noordwest ziekenhuisgroep, Alkmaar:* Ingrid van Haelst (PharmD)  *Isala, Zwolle:* Peter ter Horst (PharmD)  *Treant Zorggroep, Emmen:* Rosalie Moorlag (PharmD), Anja Vos (MD)  *Gelre ziekenhuizen, Apeldoorn/Zutphen:* Annemiek Otten-Helmers (BSc), Erik van Kan (PharmD PhD), Marije Voskamp  *St Jansdal, Harderwijk:* Marieke Ebbens (PharmD)  *Haga ziekenhuis, Den Haag:* Marieke Ezinga (PharmD), Cees van Nieuwkoop (MD), Loes Visser (PharmD)  *Rode kruis ziekenhuis, Beverwijk:* Caroline Ghazarian (PharmD), Doranne Hilarius (PharmD), Gonneke Hermanides (MD, Phd), Carlinda Bresser  *Radboud UMC, Nijmegen:* Judith Derijks-Engwegen (PharmD)  *Elisabeth Tweesteden Ziekenhuis, Tilburg:* Ebbie Boemaars (CPhT), Zahira Getrouw (PharmD), Barbara Maat (PharmD)  *Gelderse Vallei Hospital, Ede:* Peter Wierenga (PharmD)  *Maasstad ziekenhuis, Rotterdam:* Tessa Bosch (PharmD), Lisanne Krens (PharmD), Kajie Liang (MSc), Langeza Saleh (MD), Milou van Heuckelum (PharmD)  *Ikazia ziekenhuis, Rotterdam:* Lisanne Krens (PharmD)  *Tergooi Medisch Centrum, Hilversum:* Linda Hendriksen (PharmD), Paul van der Linden (PharmD)  *Dijklander ziekenhuis, Purmerend/Hoorn:* Kaylen Guda (PharmD), Kristel Crommentuijn (PharmD)  *Reinier de Graaf ziekenhuis, Delft:* Ilse Cornelissen-Wesseling (Msc), Jeroen Diepstraten (PharmD), Jacobien Ellerbroek (MD), Saskia Coenradie (PharmD)  *Zuyderland Medisch centrum, Sittard-Geleen:* Debbie Deben (PharmD), Kim Hurkens (MD), Dennis Wong (PharmD), Marion Vromen (MD), Marjolein de Bock, Suzan Savelkoul, Saskia Wolters  *Meander Medisch Centrum, Amersfoort:* Louise Andrews (PharmD), Eefje Jong (MD)  *Admiraal de Ruyterziekenhuis, Goes:* Rosanne Kranenburg (MD, Msc) |
| **Portugal**  *Hospital São Francisco Xavier, Lisboa:* Joana Soares (PharmD), Fatima Falcao (PharmD), Mariana Solano (PharmD), Erica Viegas (PharmD)  *Egas Moniz Hospital, Lisboa:* Margarida Falcao (PharmD), Helena Farinha (PharmD), Dina Mendes (PharmD), Joao Rijo (PharmD) |
| **Spain**  *Vall d'Hebron University Hospital, Barcelona:* Marta Miarons (PharmD), Maria Queralt Gorgas (PharmD)  *University Hospital Infanta Sofía, San Sebastián de los Reyes*: Cristina García Yubero (PharmD), Laura Portillo Horcajada (PharmD) |
| **Switzerland**  Luzerner Kantonalsspital: Kim Keijzers (PharmD), Silke Lim (PharmD) |
| **United Kingdom**  *Antrim Area Hospital, Antrim:* Linden Ashfield (MSc), Helen Bell (PharmD), Naomi Fitzhugh (MPharm), Glenda Fleming (PhD), Nicola Goodfellow (PhD), Joanne Hanley (BSc), Michael Scott (PhD) |

**Supplemental Table 2.** List of authors COOP research group

| **The Netherlands**  *Leiden University Medical Centre, Leiden:* Simon P. Mooijaart (MD, PhD), Jacobijn Gussekloo (MD, PhD)  *Amsterdam UMC, VUmc, Amsterdam:* Petra Elders (MD, PhD)  *Radboud University Medical Center, Nijmegen:* Geeske Peeters (PhD) |
| --- |

**Supplemental Table 3.** P-values baseline Table 1 (n = 1951)

| **Age <65 years** | | | | |
| --- | --- | --- | --- | --- |
|  | CFS 1 – 3  n = 676 (80.2%) | CFS 4 – 5  n = 112 (13.3%) | CFS 6 – 9  n = 55 (6.5%) | Overall p-value |
| **Patient characteristics**  Age (years), median [IQR]  Men, n (%) | 56 (47 – 61)  401 (59.3) | 60 (55 – 63)  67 (59.8) | 57 (52 – 62)  32 (58.2) | <0.001  0.980 |
| **Use of medication pre-admission**  Blood pressure-lowering drugs, n (%)  Antiplatelet drugs, n (%)  Oral anticoagulant drugs, n (%)  Glucose-lowering drugs, n (%)  Antipsychotic drugs and cholinesterase inhibitors, n (%)  Number of prescribed drugs, mean [IQR] | 150 (22.2)  38 (5.6)  13 (1.9)  66 (9.8)  9 (1.3)  1 (1 – 4) | 46 (41.1)  17 (15.2)  3 (2.7)  25 (22.3)  4 (3.6)  5 (1 – 8) | 21 (38.2)  9 (16.4)  4 (7.3)  8 (14.5)  2 (3.6)  4 (2 – 9) | <0.001  <0.001  0.042  <0.001  0.141  <0.001 |
| **In-hospital outcomes**  Length of stay hospital (days), median (IQR)  Intensive care admission, n (%)  Length of stay ICU (days), median [IQR]  Ventilator assisted breathing, n(%) | 5 (3 – 12)  159 (25.0)  9 (3 – 18)  124 (78.0) | 5 (3 – 8)  22 (19.6)  6 (3 – 14)  18 (81.8) | 8 (4 – 21)  23 (41.8)  9 (6 – 18)  19 (82.6) | 0.019  0.007  0.401  0.896 |
| **Destination at discharge**  Home, n (%)  Nursing home, n (%)  (Geriatric) rehabilitation, n (%) | 521 (89.8)  3 (0.5)  56 (9.7) | 83 (90.2)  0 (0)  9 (9.8) | 37 (71.2)  2 (3.8)  13 (25.0) | <0.001  0.015  0.003 |
| **Long-term outcomes**  24-months mortality, n (%) | 10 (1.5) | 10 (8.9) | 4 (7.3) | <0.001 |
| **Age ≥65 years** | | | | |
|  | CFS 1 – 3  n = 562 (50.7%) | CFS 4 – 5  n = 366 (33%) | CFS 6 – 9  n = 180 (16.2%) | Overall p-value |
| **Patient characteristics**  Age (years), median [IQR]  Men, n (%) | 74 (69 – 79)  346 (61.6) | 79 (74 – 84)  204 (55.7) | 84 (75 – 90)  91 (50.6) | <0.001  0.020 |
| **Use of medication pre-admission**  Blood pressure-lowering drugs, n (%)  Antiplatelet drugs, n (%)  Oral anticoagulant drugs, n (%)  Glucose-lowering drugs, n (%)  Antipsychotic drugs and cholinesterase inhibitors, n (%)  Number of prescribed drugs, mean [IQR] | 318 (56.6)  119 (21.1)  79 (14.1)  105 (18.7)  13 (2.3)  4 (2 – 7) | 248 (67.8)  127 (34.7)  64 (17.5)  97 (26.5)  13 (3.6)  7 (4 – 10) | 128 (71.1)  56 (31.1)  49 (27.2)  48 (26.7)  23 (12.8)  8 (5 – 11) | <0.001  <0.001  <0.001  0.007  <0.001  0.128 |
| **In-hospital outcomes**  Length of stay hospital (days), median (IQR)  Intensive care admission, n (%)  Length of stay ICU (days), median [IQR]  Ventilator assisted breathing, n(%) | 7 (4 – 15)  142 (25.6)  11 (2 – 22)  111 (78.2) | 6 (3 – 10)  51 (14.0)  4 (1 – 12)  36 (70.6) | 8 (4 – 15)  23 (13.1)  9 (3 – 13)  19 (82.6) | <0.001  <0.001  0.067  0.168 |
| **Destination at discharge**  Home, n (%)  Nursing home, n (%)  (Geriatric) rehabilitation, n (%) | 360 (73.2)  14 (2.8)  118 (24.0) | 187 (62.5)  17 (5.7)  95 (31.8) | 69 (42.1)  33 (20.1)  62 (37.8) | <0.001  <0.001  0.001 |
| **Long-term outcomes**  24-months mortality, n (%) | 46 (8.2) | 59 (16.1) | 49 (27.2) | <0.001 |

**Supplemental Table 4.** Cox proportional hazard analysis for CFS and 24-months mortality post hospital discharge(n=1885)

|  | Mortality 2 years post-discharge | | | |
| --- | --- | --- | --- | --- |
|  | Patients <65 years of age  (n=800, number of events = 21) | | Patients ≥65 years of age  (n=1085, number of events = 140) | |
|  | HR | 95% CI | HR | 95% CI |
| Model 1: univariable model |  | |  | |
| CFS |  |  |  |  |
| CFS 1 – 3 | Ref. | Ref. | Ref. | Ref. |
| CFS 4 – 5 | 8.61 | 3.28 – 22.63 | 2.38 | 1.57 – 3.59 |
| CFS 6 – 9 | 6.75 | 1.98 – 23.07 | 4.23 | 2.75 - 6.50 |
| Model 4: Model 1 + age, sex, number of drugs used, blood pressure-lowering drugs, antiplatelet drugs, oral anticoagulant drugs, glucose-lowering drugs, antipsychotic drugs, and cholinesterase inhibitors. | | | | |
| CFS |  |  |  |  |
| CFS 1 – 3 | Ref. | Ref. | Ref. | Ref. |
| CFS 4 – 5 | 4.43 | 1.60 – 1.25 | 1.48 | 0.96 – 2.27 |
| CFS 6 – 9 | 3.13 | 0.86 – 11.36 | 1.91 | 1.17 – 3.12 |
| Age | 1.09 | 0.99 – 1.18 | 1.07 | 1.05 – 1.10 |
| Woman | 0.81 | 0.31 – 2.11 | 0.70 | 0.50 – 0.99 |
| Number of drugs used | 1.25 | 1.11 – 1.40 | 1.09 | 1.04 – 1.15 |
| Blood pressure lowering drugs | 1.09 | 0.37 – 3.16 | 0.74 | 0.49 – 1.14 |
| Antiplatelet drugs | 1.24 | 0.38 – 4.03 | 1.07 | 0.70 – 1.62 |
| Oral anticoagulant drugs | 0.59 | 0.07 – 5.02 | 1.58 | 1.02 – 2.43 |
| Glucose-lowering drugs | 0.22 | 0.05 – 0.90 | 1.00 | 0.66 – 1.53 |
| Antipsychotic drugs and cholinesterase inhibitors | 0.56 | 0.06 – 5.40 | 0.68 | 0.31 – 1.49 |

**Supplemental Table 5.** Logistic regression analysis for 24-months mortality after hospital discharge (excluding in-hospital mortality) (n=1885)

|  | CFS 1 – 3 | CFS 4 – 5 | CFS 6 – 9 |
| --- | --- | --- | --- |
| Age <65 years (n = 843) | | | |
| Model 1  Model 2  Model 3  Model 4 | Ref.  Ref.  Ref.  Ref. | 6.53 (2.65 – 16.08)  5.55 (2.21 – 13.92)  3.46 (1.28 – 9.32)  3.65 (1.36 – 9.83) | 5.22 (1.58 – 17.24)  4.82 (1.45 – 16.04)  2.87 (0.81 – 10.18)  2.71 (0.75 – 9.83) |
| Age ≥65 years (n = 1108) | | | |
| Model 1  Model 2  Model 3  Model 4 | Ref.  Ref.  Ref.  Ref. | 2.16 (1.43 – 3.25)  1.58 (1.03 – 2.42)  1.33 (0.86 – 2.07)  1.34 (0.86 – 2.08) | 4.20 (2.69 – 6.55)  2.32 (1.41 – 3.82)  1.89 (1.14 – 3.15)  1.88 (1.13 – 3.15) |

Estimates are odds ratio (95% CI). CFS=Clinical Frailty Scale. Model I=crude. Model II=adjusted for sex and age. Model III=model II plus additional adjustment for the number of drugs used. Model IV=model III plus additional adjustment for blood pressure-lowering drugs, antiplatelet drugs, oral anticoagulant drugs, glucose-lowering drugs, antipsychotic drugs, and cholinesterase inhibitors.

**Supplemental Table 6.** Patient characteristics after hospital admission (including in-hospital mortality) stratified by CFS category and age (n = 2372)

P-values: provided in supplemental Table 7

|  | Age <65 years  n = 874 (36.8%) | | | Age ≥65 years  n = 1498 (63.2%) | | |
| --- | --- | --- | --- | --- | --- | --- |
|  | CFS 1 – 3  n = 689 (50.3%) | CFS 4 – 5  n = 123 (19.5%) | CFS 6 – 9  n = 62 (16.7%) | CFS 1 – 3  n = 680 (49.7%) | CFS 4 – 5  n = 509 (80.5%) | CFS 6 – 9  n = 309 (83.3%) |
| **Patient characteristics**  Age (years), median [IQR]  Men, n (%) | 56 [47 – 61]  408 (59.2) | 60 [55 – 63]  72 (58.5) | 57 [52 – 62]  37 (59.7) | 75 [70 – 80]  435 (64.0) | 80 [75 – 86]  304 (59.7) | 83 [76 – 90]  172 (55.7) |
| **Use of medication pre-admission**  Blood pressure-lowering drugs, n (%)  Antiplatelet drugs, n (%)  Oral anticoagulant drugs, n (%)  Glucose-lowering drugs, n (%)  Antipsychotic drugs and cholinesterase inhibitors, n (%)  Number of prescribed drugs, mean [IQR] | 154 (22.4)  40 (5.8)  15 (2.2)  69 (10.0)  10 (1.5)  1 [1 – 4] | 53 (43.1)  19 (15.4)  5 (4.1)  30 (24.4)  7 (5.7)  5 [1 – 8] | 24 (38.7)  9 (14.5)  4 (6.5)  10 (16.1)  4 (6.5)  5 [1.8 – 9.3] | 403 (59.3)  153 (22.5)  105 (15.4)  136 (20.0)  16 (2.4)  5 [2 – 7] | 356 (69.9)  187 (36.7)  105 (20.6)  127 (25.0)  20 (3.9)  7 [4 – 10] | 224 (72.5)  93 (30.1)  88 (28.5)  87 (28.2)  4 (1.3)  8 [5 – 11] |
| **In-hospital outcomes**  Length of stay hospital (days), median [IQR]  Intensive care admission, n (%)  Length of stay ICU (days), median [IQR]  Ventilator assisted breathing, n(%)  In-hospital mortality, n (%) | 6 [3 – 13]  167 (25.7)  10 [3 – 19]  131 (78.4)  13 (1.9) | 5 [3 – 9]  28 (22.8)  5.5 [3 – 13]  24 (85.7)  11 (8.9) | 8 [4 – 22]  28 (45.2)  8.5 [5 – 19]  22 (78.6)  7 (11.3) | 7 [4 – 16]  198 (29.5)  12 [3 – 23]  162 (81.8)  118 (17.4) | 6 [3 – 9]  73 (14.5)  5 [1- 13]  54 (74.0)  143 (28.1) | 7 [4 – 13]  44 (14.5)  8.5 [2 – 17]  36 (81.8)  129 (41.7) |
| **Destination at discharge**  Home, n (%)  Nursing home, n (%)  (Geriatric) rehabilitation, n (%) | 521 (87.9)  3 (0.5)  56 (9.4) | 83 (80.6)  0 (0.0)  9 (8.7) | 37 (62.7)  2 (3.4)  13 (22.0) | 360 (59.0)  14 (2.3)  118 (19.3) | 187 (42.3)  17 (3.8)  95 (21.5) | 69 (23.5)  33 (11.3)  62 (21.2) |
| **Long-term outcomes**  24-months mortality, n (%) | 23 (3.3) | 21 (17.1) | 11 (17.7) | 164 (24.1) | 203 (39.9) | 178 (57.6) |

**Supplemental Table 7.** P-values baseline Supplemental Table 6

| **Age <65 years** | | | | |
| --- | --- | --- | --- | --- |
|  | CFS 1 – 3  n = 689 (50.3%) | CFS 4 – 5  n = 123 (19.5%) | CFS 6 – 9  n = 62 (16.7%) | Overall p-value |
| **Patient characteristics**  Age (years), median [IQR]  Men, n (%) | 56 [47 – 61]  408 (59.2) | 60 [55 – 63]  72 (58.5) | 57 [52 – 62]  37 (59.7) | <0.001  0.986 |
| **Use of medication pre-admission**  Blood pressure-lowering drugs, n (%)  Antiplatelet drugs, n (%)  Oral anticoagulant drugs, n (%)  Glucose-lowering drugs, n (%)  Antipsychotic drugs and cholinesterase inhibitors, n (%)  Number of prescribed drugs, mean [IQR] | 154 (22.4)  40 (5.8)  15 (2.2)  69 (10.0)  10 (1.5)  1 [1 – 4] | 53 (43.1)  19 (15.4)  5 (4.1)  30 (24.4)  7 (5.7)  5 [1 – 8] | 24 (38.7)  9 (14.5)  4 (6.5)  10 (16.1)  4 (6.5)  5 [1.8 – 9.3] | <0.001  <0.001  0.090  <0.001  0.002  0.151 |
| **In-hospital outcomes**  Length of stay hospital (days), median (IQR)  Intensive care admission, n (%)  Length of stay ICU (days), median [IQR]  Ventilator assisted breathing, n(%)  In-hospital mortality, n (%) | 6 [3 – 13]  167 (25.7)  10 [3 – 19]  131 (78.4)  13 (1.9) | 5 [3 – 9]  28 (22.8)  5.5 [3 – 13]  24 (85.7)  11 (8.9) | 8 [4 – 22]  28 (45.2)  8.5 [5 – 19]  22 (78.6)  7 (11.3) | 0.032  0.002  0.215  0.579  <0.001 |
| **Destination at discharge**  Home, n (%)  Nursing home, n (%)  (Geriatric) rehabilitation, n (%) | 521 (87.9)  3 (0.5)  56 (9.4) | 83 (80.6)  0 (0.0)  9 (8.7) | 37 (62.7)  2 (3.4)  13 (22.0) | <0.001  0.023  0.009 |
| **Long-term outcomes**  24-months mortality, n (%) | 23 (3.3) | 21 (17.1) | 11 (17.7) | <0.001 |
| **Age ≥65 years** | | | | |
|  | CFS 1 – 3  n = 680 (49.7%) | CFS 4 – 5  n = 509 (80.5%) | CFS 6 – 9  n = 309 (83.3%) | Overall p-value |
| **Patient characteristics**  Age (years), median [IQR]  Men, n (%) | 75 [70 – 80]  435 (64.0) | 80 [75 – 86]  304 (59.7) | 83 [76 – 90]  172 (55.7) | <0.001  0.038 |
| **Use of medication pre-admission**  Blood pressure-lowering drugs, n (%)  Antiplatelet drugs, n (%)  Oral anticoagulant drugs, n (%)  Glucose-lowering drugs, n (%)  Antipsychotic drugs and cholinesterase inhibitors, n (%)  Number of prescribed drugs, mean [IQR] | 403 (59.3)  153 (22.5)  105 (15.4)  136 (20.0)  16 (2.4)  5 [2 – 7] | 356 (69.9)  187 (36.7)  105 (20.6)  127 (25.0)  20 (3.9)  7 [4 – 10] | 224 (72.5)  93 (30.1)  88 (28.5)  87 (28.2)  4 (1.3)  8 [5 – 11] | <0.001  <0.001  <0.001  0.011  <0.001  <0.001 |
| **In-hospital outcomes**  Length of stay hospital (days), median (IQR)  Intensive care admission, n (%)  Length of stay ICU (days), median [IQR]  Ventilator assisted breathing, n(%)  In-hospital mortality, n (%) | 7 [4 – 16]  198 (29.5)  12 [3 – 23]  162 (81.8)  118 (17.4) | 6 [3 – 9]  73 (14.5)  5 [1- 13]  54 (74.0)  143 (28.1) | 7 [4 – 13]  44 (14.5)  8.5 [2.3 – 16.5]  36 (81.8)  129 (41.7) | <0.001  <0.001  0.007  0.123  <0.001 |
| **Destination at discharge**  Home, n (%)  Nursing home, n (%)  (Geriatric) rehabilitation, n (%) | 360 (59.0)  14 (2.3)  118 (19.3) | 187 (42.3)  17 (3.8)  95 (21.5) | 69 (23.5)  33 (11.3)  62 (21.2) | <0.001  <0.001  0.655 |
| **Long-term outcomes**  24-months mortality, n (%) | 164 (24.1) | 203 (39.9) | 178 (57.6) | <0.001 |


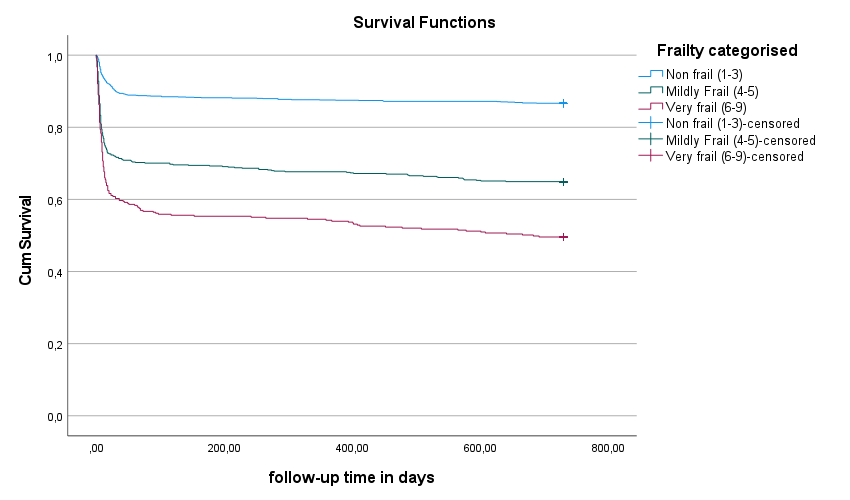


**Legend**


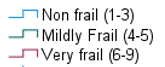


**Supplemental Figure 1.** 24-months mortality after hospital admission (including in-hospital mortality) (n =2311)

**Supplemental Table 8.** Cox proportional hazard analysis after hospital admission (including in-hospital mortality)

|  | CFS 1 – 3 | CFS 4 – 5 | CFS 6 – 9 |
| --- | --- | --- | --- |
| Age <65 years (n = 835, number of events = 52) | | | |
| Model 1  Model 2  Model 3  Model 4 | Ref.  Ref.  Ref.  Ref. | 6.06 (3.28 – 11.17)  4.80 (2.58 – 8.95)  3.11 (1.60 – 6.06)  3.23 (1.65 – 6.33) | 6.21 (2.98 – 12.97)  5.50 (2.62 – 11.52)  3.57 (1.66 – 7.70)  3.36 (1.54 – 7.33) |
| Age ≥65 years (n = 1476, number of events = 529) | | | |
| Model 1  Model 2  Model 3  Model 4 | Ref.  Ref.  Ref.  Ref. | 1.96 (1.59 – 2.42)  1.55 (1.25 – 1.93)  1.40 (1.12 – 1.74)  1.41 (1.13 – 1.76) | 3.18 (2.56 – 3.95)  2.11 (1.66 – 2.68)  1.85 (1.45 – 2.35)  1.84 (1.44 – 2.34) |

Estimates are hazard ratios (95% CI). CFS=Clinical Frailty Scale. Model I=crude. Model II=adjusted for sex and age. Model III=model II plus additional adjustment for the number of drugs used. Model IV=model III plus additional adjustment for blood pressure-lowering drugs, antiplatelet drugs, oral anticoagulant drugs, glucose-lowering drugs, antipsychotic drugs, and cholinesterase inhibitors.

**Supplemental Table 9.** Analysis comparing patients with and without a CFS

|  | CFS sample  N = 2372 (83.3%) | Non-CFS sample  N = 476 (16.7%) | p-value |
| --- | --- | --- | --- |
| **Patient characteristics**  Age (years), median [IQR]  Male, n (%) | 71 (60 – 80)  1482 (60.2) | 72 (61 – 80)  303 (63.7) | 0.765  0.165 |
| **Use of medication pre-admission**  Blood pressure-lowering drugs, n (%)  Antiplatelet drugs, n (%)  Oral anticoagulant drugs, n (%)  Glucose-lowering drugs, n (%)  Antipsychotic drugs and cholinesterase inhibitors, n (%)  Number of prescribed drugs, mean [IQR] | 1214 (51.2)  501 (21.2)  322 (13.6)  459 (19.4)  101 (4.3)  5 (2 – 8) | 265 (55.8)  118 (24.8)  59 (12.4)  100 (21.1)  25 (5.3)  5 (2 – 9) | 0.070  0.077  0.555  0.411  0.329  0.003 |
| **In-hospital outcomes**  Length of stay hospital (days), median (IQR)  Intensive care admission, n (%)  Length of stay ICU (days), median [IQR]  Ventilator assisted breathing, n(%)  In-hospital mortality, n (%) | 6 (3 – 13)  538 (23.2)  9 (3 – 19)  429 (79.7)  421 (17.7) | 7 (3 – 13)  110 (25.1)  10 (4 – 18)  88 (80.0)  104 (22.3) | 0.096  0.390  0.537  0.532  0.021 |
| **Destination at discharge**  Home, n (%)  Nursing home, n (%)  (Geriatric) rehabilitation, n (%) | 1257 (59.9)  69 (3.3)  353 (16.8) | 230 (55.6)  24 (5.8)  56 (13.5) | 0.104  0.013  0.098 |
| **Long-term outcomes**  24-months mortality, n (%) | 1773 (74.7) | 328 (68.9) | 0.008 |

Missing values: CFS sample: 56 Length of hospital stay, 56 ICU admission, 9 ICU length of stay, 18 ventilator assisted breathing, 272 Destination of discharge. Non-CFS sample: 1 Blood pressure-lowering drugs, 1 Antiplatelet drugs, 1 Oral anticoagulant drugs, 1 Glucose-lowering drugs, 1 anti-psychotic drugs and cholinesterase inhibitors, 1 number of prescribed drugs, 44 Length of hospital stay, 38 ICU admission, 6 ICU length of stay, 8 Ventilator assisted breathing, 9 In-hospital mortality, 62 Destination of discharge.

**Supplemental Table 10.** Analysis comparing patients in the Netherlands and other countries

|  | The Netherlands  N = 1979 (83.4) | Other countries  N = 393 (16.6) | p-value |
| --- | --- | --- | --- |
| **Patient characteristics**  Age (years), median [IQR]  Male, n (%) | 72 (61 – 80)  1211 (61.2) | 68 (54 – 79)  217 (55.2) | <0.001  0.027 |
| **Use of medication pre-admission**  Blood pressure-lowering drugs, n (%)  Antiplatelet drugs, n (%)  Oral anticoagulant drugs, n (%)  Glucose-lowering drugs, n (%)  Antipsychotic drugs and cholinesterase inhibitors, n (%)  Number of prescribed drugs, mean [IQR] | 1017 (51.4)  437 (22.1)  287 (14.5)  387 (19.6)  58 (2.9)  5 (2 – 8) | 197 (50.1)  64 (16.3)  35 (8.9)  72 (18.3)  43 (10.9)  3 (1 – 7) | 0.647  0.010  0.003  0.571  <0.001  <0.001 |
| **In-hospital outcomes**  Length of stay hospital (days), median (IQR)  Intensive care admission, n (%)  Length of stay ICU (days), median [IQR]  Ventilator assisted breathing, n(%)  In-hospital mortality, n (%) | 6 (3 – 11)  459 (23.3)  9 (2 – 20)  378 (84.6)  360 (18.2) | 11 (5 – 22)  79 (22.8)  11 (6 – 19)  51 (69.9)  61 (15.5) | <0.001  0.849  0.162  0.002  0.206 |
| **Destination at discharge**  Home, n (%)  Nursing home, n (%)  (Geriatric) rehabilitation, n (%) | 1032 (57.5)  66 (3.7)  337 (18.8) | 225 (73.8)  3 (1.0)  16 (5.2) | <0.001  0.020  <0.001 |
| **Long-term outcomes**  24-months mortality, n (%) | 1466 (74.1) | 307 (78.1) | 0.092 |

Missing values: the Netherlands: 7 Hospital length of stay, 9 ICU admission, 2 ICU length of stay, 12 Ventilator assisted breathing, 184 Destination at discharge. Other countries: 49 Hospital length of stay, 47 ICU admission, 7 ICU length of stay, 6 Ventilator assisted breathing, 88 Destination at discharge.
